# Mobile Sensing Systems

**DOI:** 10.3390/s131217292

**Published:** 2013-12-16

**Authors:** Elsa Macias, Alvaro Suarez, Jaime Lloret

**Affiliations:** 1 Grupo de Arquitectura y Concurrencia (GAC), Departamento de Ingeniería Telemática, Universidad de Las Palmas de Gran Canaria, Campus Universitario de Tafira, Las Palmas de Gran Canaria (Gran Canaria) 35017, Spain; E-Mail: asuarez@dit.ulpgc.es; 2 Integrated Management Coastal Research Institute, Universidad Politécnica de Valencia, C/Paranimf, n° 1, Grao de Gandia 46730, Spain; E-Mail: jlloret@dcom.upv.es

**Keywords:** individual mobile sensing, crowd sensing, mobile operating systems, mobile cloud, smart phone, sensors, ubiquitous sensing, web sensing

## Abstract

Rich-sensor smart phones have made possible the recent birth of the *mobile sensing* research area as part of ubiquitous sensing which integrates other areas such as *wireless sensor networks* and *web sensing*. There are several types of *mobile sensing*: individual, participatory, opportunistic, crowd, social, *etc*. The object of sensing can be people-centered or environment-centered. The sensing domain can be home, urban, vehicular… Currently there are barriers that limit the social acceptance of mobile sensing systems. Examples of social barriers are privacy concerns, restrictive laws in some countries and the absence of economic incentives that might encourage people to participate in a sensing campaign. Several technical barriers are phone energy savings and the variety of sensors and software for their management. Some existing surveys partially tackle the topic of mobile sensing systems. Published papers theoretically or partially solve the above barriers. We complete the above surveys with new works, review the barriers of mobile sensing systems and propose some ideas for efficiently implementing sensing, fusion, learning, security, privacy and energy saving for any type of mobile sensing system, and propose several realistic research challenges. The main objective is to reduce the learning curve in mobile sensing systems where the complexity is very high.

## 1. Introduction

Ubiquitous Computing is a research area that has significantly changed the way computing and communication resources are used nowadays. Reeves [1] affirmed that Ubiquitous Computing has some evident possibilities that will appear attractive to various stakeholders in public and private sectors: there are enabling technologies in our days and in the near future, projection of technology in Society and these technologies can be potentially embedded everywhere, leading to a proliferation of different technologies and services. Examples of enabling ubiquitous technologies are: (a) *Wireless Sensor Networks* (*WSNs*) embedded in people, furniture, homes, urban, rural, abovewater, underwater…; (b) *Sensor Webs* [2] that aggregate sensed data of geographically distributed robots, satellites, ships, airplanes…; (c) Rich-sensor smart phones and related mobile computing devices which have been used for *Social Sensing* [3]. The combination of WSN, rich-sensor smart phones and Web results in a powerful ubiquitous computing platform [4].

A *Mobile Sensing System* (*MSS*) requires a user level *Application* (*App*) running on the phone for reading an internal phone's sensor, or external sensors in the WSN and reporting sensed data to the Web. To do this, the phone operating system must offer an *Application Programming Interface* (*API*) to manage the data reading and reporting.

The *App* of an *individual* MSS only runs on one phone that does not cooperate with other *Apps* installed in other phones. In a *participatory* MSS [5] the *App* is executed on several phones in a distributed manner and a sensing campaign is organized in order the phone's users (*participants*) to sense disjoint parts of the area to be sensed. There is a sensing campaign administrator that is in charge of organizing the entire campaign. In a *crowd* MSS a requester can start a campaign using his phone [6] or a large number of different MSS *Apps* can be coordinated in order to avoid redundant sensing in a determined area [7]. A type of crowd sensing is *place-centric sensing* [8]. In *opportunistic* sensing, participants try their best to participate full or part time. The last three kinds of MSS use a server, normally accessible via the Internet. The data contributed by multiple participants are combined (integrated) in this server to build views of sensed data or to elaborate statistics.

The application domain of a MSS depends on the location of the phone. It could be personal (*healthcare sensing*), vehicular (*vehicular sensing*), home (*smart homes*), urban (*urban sensing*) or city (*smart cities*), rural, a local geographic area or a global geographic area… The role of a person in urban sensing should be opportunistic [9]. Depending on the object of sensing a MSS could collect data about people (*people-centric*) or about the environment (*environment-centric*). A MSS can implement sensing processes continuously, trigged by a user action, upon receiving a message of another participant… Continuous MSS presents several key research challenges: (a) since a MSS relies on volunteer participants, consequently, collected samples are typically randomly distributed in space and time, and are incomplete; (b) learning techniques must be used to infer participant context and activities. Recently, Xu *et al* [10] applied unsupervised learning in the phone using microphone sensor to count the number of people speaking; (c) participant privacy must be preserved [11,12]; (d) evaluation of trustworthiness of the sensed data; (e) assure the phone energy conservation. The LittleRock project [13] proposed a low-power co-processor to manage sensors' events. The optimal clock frequency and entering low-power standby mode between frames was used for image sensing in [14]; (f) coverage control and the particular mobility patterns of the participants. In [15] some initial ideas are presented about controlling the movement of participants avoiding driving them into areas with little to no sensing coverage, allowing for greater coverage with fewer participants and higher densities of measurements in a specific region; (g) due to sensing, the highly skewed spatial-temporal sensing frequency [16] depends on the phones that will participate in the sensing process; and (h) stimulation of participation [17] because, *a priori*, participants are doing philanthropic work.

In the published surveys several examples of MSS have been presented. There is a plethora of MSS: some of them are commercial, others are only a proof-of-concept, and others are systems that have only been tested in research laboratories. The typical example of people-centric MSS is healthcare, which uses continuous sensing for serious diseases, or monitoring people [18]. Typical examples of environment-centric MSS are, among others, catastrophes, traffic monitoring, collaborative weather prediction, noise level monitoring in cities and air pollution. There are mobile applications that use the combination of different sensors [19,20]. Each published survey about MSS reviews a number of systems [5,21,22], but there are many MSS that have not been reviewed in those surveys. Now we present some of them.

People-centric *Apps* such as *EyePhone* [23] infer the position of the eye on the phone display and lets the user activate the phone with the eyes. *WakeNsmile* [24] detects when a sleeper was most likely to wake up with a smile and hopefully in a better mood. *SoundSense* [25] classifies the ambient sound, music, speech and learns new sound events in different users' daily life. *WalkSafe* [26] implements a classification pipeline to help people cross roads while they are walking and talking. That *App* alerts the user of a potentially unsafe situation using sounds and vibrations from the phone.

Some sensing *Apps* use location sensors. *Apps* such as *LifeMap* [27] provide indoor location information combining the information with *Wireless Fidelity* (*WiFi*), and *Global Positioning System* (*GPS*) to generate user context in daily life. *BeepBeep* [28] is a high-accuracy ranging and localization library that *Windows Mobile 5.0* applications can load and use. For example, the *Draw-In-The-Air* (*DITA*) *App* used the *BeepBeep* library to turn the device on or off with a tickle or cross gesture and a left-leaning or right-leaning gesture to switch to the previous or next channel. The *Point&Connect App* used *BeepBeep* to pair a user's phone with another nearby device by making a simple hand gesture to point her phone towards the intended target. Android phones are used in [29] for semantic localization such as points of interest, street or city names. Authors showed that the learning of different pedestrian movement types, such as walking and running, requires more sensing information, for example from an accelerometer. Semantic data segments can be designed to classify the user's activities such as sitting, standing and running using the accelerometer sensor [30]. *ConceptNet* [31] is a semantic network designed for commonsense contextual reasoning to be applied to a specific sensor fusion problem involving both user location and activity. The sensed data from GPS, Google Maps, camera and accelerometer are classified, labeled and fused together on a common sense basis.

In the healthcare domain an *App* was considered a gateway between the medical devices and the Web using Bluetooth and WiFi [32]. *Mobile Ultra Violet Monitor App* [33] processed the UV radiation level measured by a handheld sensor and displayed the post-processed data to the user to limit overexposure.

A prototype of participatory MSS applied to bus arrival time prediction based on bus passengers' participation was presented in [34]. It solely relied on the participants and it was independent of the bus operating companies. *ParkSense* [35] is an *App* that tries to help car drivers to find parking spots. The developers studied the consumption of different sensors of the phone in order to optimize their use and presented a survey of related works. *CoenoFire* [36] is a participatory MSS for improving firefighter work in real scenarios. The sensing campaign is organized by the fire chiefs and sensed data are shared on a server. A participatory MSS similar to *Bikenet* [37] was presented in [38]. They used an external sensor located in a bike for acquiring periodic CO and CO_2_ measurements and sending values to an Android phone. Bluetooth connection was used between the *App* and the sensor. Then, sensed data were compressed and transmitted to a monitoring server using a specific WiFi *Access Point* (*AP*) available on the bike sharing racks. The monitoring server validated and interpolated data acquired by bikers and produced urban pollution level maps. There was a feedback since the biker could know the healthiest route. CO pollution was analyzed in [39] using a Bluetooth connection between a sensor located in a car and the phone and commercial servers to share tasks among social sensing participants.

The platform for 3D map-based visualization on mobile devices presented in [40], powered by augmented reality named ARCAMA-3D, offered context-aware interactions to enable users to navigate in an area with their mobile devices and interactively discover their surroundings. The system integrated real-time GPS and other embedded sensors and exploited user's context and preferences in order to provide him/her with the necessary information. In return, the user consults the information such as text, photo, audio or video files that were published on the 3D model with the help of an augmented reality technique.

An *Ambient intelligence* approach can be used to implement continuous patient monitoring for improving the communication between patients and doctors [41]. This approach can be used to automatically generate individual patients' profiles, self-control and education modules for their chronic diseases.

*Social sensing* includes social interactions such as co-located, face-to-face interaction, excluding electronically mediated interactions such as chat, social network activity and other kinds of electronic communication. *Crowd*++ [10] is an *App* for counting people in different geographical scenarios. The phone camera can be used to measure body orientations [42]. The accelerometer can be used to detect chest wall vibrations and hence speech activity. WiFi can be used for distance estimation. The challenges of social sensing [43] and a middleware, such as *Comm2Sense* [44] that uses the WiFi signal to detect physical proximity have been presented.

A survey about some future applications of mobile sensing was presented in [45]; it reviews different ways to do mobile sensing, the challenges of mobile sensing and the use of Web services for mobile sensing and introduced social and community aware intelligence. In [46], this concept was refined as the application of mobile sensing, Web sensing and other kinds of ubiquitous sensing techniques to society. In this context, *Community Similarity Networks* [47] can be used to implement efficient groups for participatory sensing. A step beyond this work is the concept of Guo *et al.* [48] named *embedded intelligence* (“*the knowledge learned from human-IoT interaction which refers to the knowledge about human life, ambient dynamics, and social connection/interaction*”). They present the three dimensions of embedded intelligence: user, ambient and social; and the architecture of embedded intelligence applications. In this architecture, the lowest level is built on the *Internet of Things* (*IoT*) a term which allows sensing devices to register digital traces of people-environment interactions. Examples of devices are mobile phones which can be used to discover individual and social information of people (*social intelligence*).

We focus our work on MSS that potentially interoperates with WSN using a communication middleware and Web services. This kind of system is complex to implement efficiently. There are several research challenges like saving the phone's battery energy, privacy and security. A cloud computing infrastructure can be used to treat with the complexity of fusion or calculation of thing's context. If the participants meet themselves, it will be recommendable to use social network software to allow richer patterns of social participation [49].

As shown a large number of works have been published and a considerable number of mobile sensing applications developed. The learning curve in mobile sensing could be very large. For this reason, in this paper we present challenges and solutions for practical MSS. Then, we present an practical MSS architecture proposal considering not only technical, but also social issues.

The remainder of the paper is organized as follows: in Section 2 we review sensors and software for their management in order to establish realistic assumptions that will help us to present the ideas of a practical MSS. Section 3 presents important design issues for MSS: fusion and learning, security and privacy, and saving energy. Our ideas to design MSS taking into account current research trends and presenting research challenges are discussed in Section 4. Finally, in Section 5, we draw our main conclusions and describe future work.

## 2. Mobile Sensors and Their Software

This section is devoted to reviewing the sensors available on most smart phones, the operating system software for managing the sensors to do mobile sensing, and some preliminary works that use sensors and the appropriate software to do mobile sensing.

Most mobile phones include the following sensors: *GPS* for outdoors localization, an *Accelerometer* S*ensor* (*AS*) to measure acceleration, a *Compass Sensor* (*CS*) to determine the angle by which the phone is rotated relative to the Earth's magnetic North Pole, a *Gyroscope Sensor* (*GS*) to measure the angular rate of how quickly the object turns, an *Image Sensor* (*IS*) to capture images and record videos, an *Ambient Light Sensor* (*ALS*) to detect how much luminance is present, a *Proximity Sensor* (*PS*) to detect how close the phone is to the user's body, *Touch* S*ensors* (*TS*) to detect the presence and location of a touch with a finger or stylus pen within the display area, and a *Temperature Sensor* (*TS*), *Humidity Sensor* (*HS*) and *Atmospheric Pressure Sensor* (*APS*) to detect real-time environmental temperature, humidity and atmosphere pressure, respectively. *AS*, *CS*, *GS* and *GPS* are mainly used in mobile sensing.

Mobile operating systems provide an *API* to manage phone's sensors. They are summarized in Table 1. *BlackBerry OS* exports a Java™ development environment that includes a BlackBerry IDE, a phone simulator, and API for *Java 2 Micro Edition* (*J2ME*) Platform.

The *Windows***™***Mobile*® operating system provides *Microsoft.Devices.Sensors* and *System.Device. Location* namespaces for accessing sensors. The complete toolset *Qt framework* [50], available for *Symbian* phones and the Nokia N9, provides the *Qt Mobility Language* (*QML*), a JavaScript based declarative language. The *QtMobility* Project includes the API shown in Table 1.

The Android [51]*Software Development Kit* (*SDK*), which includes an emulator of a run time environment for testing and debugging, exports classes and interfaces of the *Android Sensor Framework* in Java language. The *sensor framework* is part of the *android.hardware* package.

The iOS SDK [52], Xcode and Interface Builder export classes of the Core Motion Framework in Objective C language.

There is not a common API to program context aware mobile applications.

Access to external sensors is traditionally managed with very different APIs and this heterogeneity leads to interoperability problems among sensors [53]. One solution is a common API for external sensors and internal sensors in the phone [54], or the implementation of a complex mechanism that allows interoperation among external and phone's sensors [55].

Android and *iCore Windows 8 App* developers can build applications on top of the sensor fusion software [56], developed by Kionix Inc., that combines inputs from an *AS*, magnetometer and *GS* into synthetic sensors, that is to say, a fusion of different physical sensors' values in order to provide high level semantic information such as orientation, rotation vector, linear acceleration, and gravity.

The API designed in [57] was able to manage several sensors to program context-aware mobile applications. It was based on a tuple space model (*LinuxTuples*) that used a blackboard to process the inputs of the sensors.

The *Open Data Kit* [58] provided a high level framework and driver construction tools to facilitate the connection between internal and external sensors. The user must build the sensor driver to manage the communication between the external sensors and the phone.

There are some proposals limited to Nokia S60 devices using Qt. An initial work in which a cross- platform environment is used to program mobile devices with different sensors is described in [59]. In [60], the authors presented a pre-alpha platform and an API [61] for accessing a phone's sensors as well as external sensors that can be accessed using Bluetooth or other compatible wireless technology. It allows defining synthetic sensors.

An early initiative to standardize the access to mobile sensors is the Mobile sensor API [62] for J2ME standardized by the *Java Specification Report* (*JSR*) 256 [63]. It uses *MIDlets* to fetch and monitor sensed data from internal and external sensors uniformly. There are several examples of healthcare applications that use JSR256: (a) Remote monitoring of the activity characteristics of elderly patients in home or community sensing using the AS [64]; (b) determine when the activity of elderly people is being undertaken by a subject carrying the handset and to quantify the activity level [65] and (c) Accessing external healthcare sensors in the body of patients and reporting the sensed values to a remote telemedicine server [66]. Cecilio *et al.* [67] argued that JSR 256 was not appropriate for considering heterogeneous external sensors and did not address the reconfiguration of external interconnected sensor/actuators.

*StreamImput* [68] by the *Khronos Group Industry Consortium* is expected to be an API that enables applications to discover and use new generation sensors in order to create sophisticated user interactions. To the best of our knowledge this library has not been released for mobile phones.

Mobile tasking applications that process continuous data from multiple sensors can use in the future the *Code In The Air* (*CITA*) system [69]. Users can easily compose their own tasks by mixing and matching available activity primitives or tasks (e.g., *isWalking*, *isBiking*, *isRunning*, *isDriving*, *isOutdoors*, *enterPlace*, and *leavePlace*) and the conditions and actions to perform.

*PhoneGap* [70] has been used to create mobile applications for iOS, Android, Blackberry, Windows Phone, Palm WebOS, Bada and Symbian using *HyperText Markup Language* (*HTML*), *Cascading Style Sheet* (*CSS*) and *Javascript*. Regarding sensor programming, *PhoneGap* provides an API to capture mobile phone motion in the X, Y, and Z directions, access the audio, image, video and compass, and the latitude and longitude without guaranteeing that the API returns the device's current location. The programming of low level actions in *PhoneGap* is currently very limited.

## 3. Design Issues of Mobile Sensing Systems

Continuous sensing using GPS can lead to privacy and phone energy consumption problems. If a security mechanism is computationally intensive, the battery will drain rapidly. In this section we review some works which study fusion and learning, security and privacy and energy saving inside a phone or with external sensors that do not belong to a WSN. These works are not reviewed in previous published surveys.

### 3.1. Fusion and Learning

Fusion can be used to design context aware applications. The context of an object is any information that characterizes its state and allows for dynamic changes in applications and/or the automatic invocation of new applications [71]. Context information is derived from these sources: physical internal and external sensors, devices in the environment, and data sources on the user's device or accessible via the telecoms or Internet infrastructure. Two fusion examples are: (a) an earbud to read a person's blood oxygen level, body temperature, heat flux and heart rate, and a heart-rate monitor that can be embedded into a device like an iPhone to identify a user or determine the user's mood [72]; (b) InvenSense's MPU-9150 is the world's first 9-axis MotionTracking [73] that fuses data from the three-axis of AS, CS and GS. *Indoor Navigation Systems* (*INS*) can be enriched with the fusion of built-in sensors and external inputs such as a map of the floor, a GPS/cell based positioning, or a WiFi fingerprinting [74–76]. Indoor Android phone localization can be achieved fusing several sources [77] such as AS, magnetometer, GS and RSSI of several WiFi AP.

Learning can be used for inferring the current activities of mobile users [78]. The authors inferred actions like walking, cooking, reading, driving and eating, using the data sensed by the mobile phone and a hidden Markov model. The user's intervention was needed to label the beginning and the end of each activity. Chen *et al.* [79] showed preliminary ideas about a framework to analysis and infer human behavior patterns fusing raw WiFi RSSI readings and AS to extract fine grained significant locations in user's daily life. Two learning examples are: (a) Koukoumidis *et al.* [80] who used IS to collaboratively detect and predict the schedule of traffic signals using an iPhone in cars instead of the traditionally used sources such as GPS, AS or single axis GS; (b) *Imsec* [81] is an Android proof-of-concept application which can securely capture, store, and transfer phone camera-generated images in a war zone.

We briefly summarize in Table 2 the characteristics of the above reviewed systems and applications according to the following items: it is a realistic implementation (application) or it is a start point for app developers, mobile operating systems supported, used sensors, details about how the fusion is made (used method, applied technique, *etc.*), and some application domains.

### 3.2. Security and Privacy

System security must avoid denial of service, eavesdropping and illegal physical accesses at the sensing server. Information privacy must be assured implementing data encryption, data integrality, authentication and freshness protection. The use of the phone's GPS is normally authorized by the phone user. But GS and AS can be used by third party applications in order to infer the location of touch-screen taps without the permission of the phone user. The work presented in [82] provides a simple example where privacy is a barrier to the acceptance of mobile sensing. It uses a framework which includes motion sensor readings, accelerometer and gyroscope sensor data combined with machine learning analysis.

Trustworthy uploading of sensed data to the sensing server is a challenge. Sensors are well calibrated, but humans are less reliable. That is, people must be well educated in the correct use of sensors. Algorithm design for obtaining the optimal solution to the discovery of the truth information reported by different participants is important. This happens when near participants in a social sensing campaign report different data about the same experiment. A theoretical algorithm presented in [83] could be put into practice due to its potential relevant applications.

Security and privacy have also a social aspect: intellectual property laws and other important social legislation are another barrier [84]. In that work three foundational design principles are discussed: primacy of participants, data legibility, and engagement of participants throughout the data life cycle. The expansion of the *codes of fair information practice to protect privacy* in MSS is proposed. This is a very important topic in MSS because in several countries, laws impose a lot of restrictions on social sensing.

Security is a technical barrier and privacy is a personal barrier in telemedicine [85]. In participatory MSS, privacy is the guarantee that participants maintain control over the release of their sensitive information, including the protection of information that can be inferred from both the sensor readings as well as the interaction of the users with the participatory MSS [86]. Each component of the participatory MSS has its own responsibilities for assuring privacy: (a) Sensors: participants that report daily their GPS and AS movement tracking could easily be identified. To avoid this, the frequency of reporting could be varied for example, to report the position only when the street changes; (b) Administrator of sensing campaigns: A malicious administer can identify a lot of critical information about participants; (c) Reporting: If a participant does not use an anonymous proxy for example, his reported data will be easily identified knowing its *Internet Protocol* (*IP*) Address; (d) Data processing on the server: The best practice is to aggregate participants' sensed data to avoid participant identification; (e) Social networks: Normally pictures and audios easily identify a participant. In [87] a good introductory review of data mining and data aggregation is presented. The authors also categorized solutions using some parameters like perturbation, k-anonymity, secure multi-party computation and homomorphism encryption.

There are technical solutions for MSS security and privacy. Some solutions are theoretical proposals or obtained via simulation [87–89]. An experimental approach is presented in [87], and others have proposed Android applications [90–92]. The sensing domain is a general one in [87,88] and specific for urban sensing in [89]. Healthcare is the sensing domain of [90–92].

The hiding of which phone matches a query and which data is sensed is a way of implementing privacy [89]. A large-scale system resembling a mesh network of sensors is assumed. An inconvenience for mobile phones is that they only could be connected to this mesh infrastructure using WiFi. There is a tradeoff between sensing accuracy and privacy. This tradeoff is related to the number of dimensions, categories, and participants [87]. The server reconstructs the probability density functions of the original distributions using the sensed values, but without knowing the participants' actual data. These theoretical ideas must be put in practice to observe results respecting real world scenarios. In [90–92] the authors focused on the safe processing of the phone sensed data in that mitigating attacks by malware and other attacking software is an important challenge. In [90,91] GPS, IS and microphone were used and in [92] external sensors connected to the phone via *Bluetooth* and *ZigBee* were used. The problem analyzed in [90,91] is trustworthiness: how to verify that authenticity and fidelity are achieved. Confidentiality and integrity properties are analyzed between the outer sensors and the phone [92]. All the reviewed works need to verify that the running Android application is safe and differ in the way they test privacy: the first two works used *TaintDroid* [93] to trash the dependencies among data and applications in order to register possible alterations of data. This is a very cheap solution which does not include additional hardware; perhaps they should consider including the modern *Trust Computing Platforms* incorporated in recent commercial phones. On the contrary, [92] requires additional, expensive and sophisticated mechanisms that can limit its applicability. That is, each sensor had a cryptographic key which is known to the *Secure Data* (*SD*) card which has a key also known to the outer sensors. The key distribution is done directly between the SD, without the intervention of the phone and the outer sensors. In this way, the malware will not be aware of the keys. Among the seven limitations they exposed, one curious limitation was that data cannot be displayed to the patient because it would be vulnerable to malware. They suppose the outer sensors communicate with the phone using *Zigbee*, *Bluetooth* or its secure protocol, so in practice they can only directly use *Bluetooth* technology, and support its secure protocol over it, because *Zigbee* is not directly supported in present phones. Trusted computing platform is used in recently released phones.

### 3.3. Energy Saving

Energy saving is a very important issue in mobile *App* design and implementation. A kernel module of the modern smart phone's operating system manages it using energy profilers. For example, *Eprof* [94] considered that optimizing energy consumption is of critical importance and it was the first fine-grained energy profiler for phone *Apps*. It was implemented on *Android* and *Windows Mobile*. The aim of *Eprof* was guessing where the energy was spent inside any App, for example in storage [95].

Energy saving is a key issue for continuous sensing because the phone battery drains rapidly [96]. For this reason, the main objective of energy saving in continuous sensing is to control the actions of sensors and suspend them when necessary. To do this, three different kind of sensors were identified in [97]: (a) Basic: sensors that work continuously, for example cell identification sensors; (b) Light-Duty: software-based sensors that do not consume too much energy; (c) Heavy-Duty: sensors that are not necessarily always on, for example GPS and microphone.

Energy saving has mainly been studied in theoretically the past, but in [98] a report in the healthcare domain was presented which explained the lessons learnt after their system was tested with several people for a long time.

There are several approaches to control energy saving. Among them we review Green Technology and specific middlewares for energy saving. In this context energy saving in mobile devices follows three directions [99]: (a) Energy profiling which satisfies quality of service and quality of experience; (b) Utilization of multiple radio switching to save energy still remains challenging; (c) Effective transmissions: mobile applications and services can include inherent power-saving designs, predication- based adaptation by learning the historical pattern, and proxy-based caching.

The optimization of the sensor duty cycles was studied in [97,100] using different mathematical models. A Markov chains model was formulated [100] for minimizing the expected user state estimation error, while maintaining an energy consumption budget. The results were numerically compared against uniform periodic sampling and they found that the performance gains depend upon the user state transition probabilities. Machine learning algorithms performing offline training of the inference models were used to observe the value stability provided by the sensors in order to disconnect them for some time. During that time interval, last read values were used. The calculation of the intervals of time in which to use the last read values of the sensors in order to optimize the energy saving is challenging. These theoretical models must be verified with real scenario experiments because there are several issues that influence energy consumption. Those issues can be taken into account theoretically, for example, the sporadic variation of the wireless channel in the presence of obstacles.

AS sampling frequency *versus* sensed data accuracy impacts energy savings. This affects human activity recognition. For example, [101] showed a sequence of moderately-long lasting activities, and many of these commonplace activities can be classified quite accurately, without requiring sophisticated features or high sampling rates.

The main objective of energy saving middleware is to accommodate the energy consumption taking into account real world problems and minimizing its energy consumption overhead. In [102], *Acquisitional Context Engine* (*ACE*) middleware observes the behavior of the participant in different physical contexts (home, driving a car, in office…) and correlates sensor values that could define the location (context attributes) in which the user could be. It dynamically learned relationships among various context attributes and basically used *inference caching* for opportunistically inferring one context attribute and try to do speculative sensing. In [103], Lee *et al.* designed an energy saving middleware applied to close participants avoiding them the necessity to repeat the sensing process over a shared geographical area. Group formation, the distribution of sensing planning, and the mobility of participants were identified as challenges. An identified barrier was that although the participants were closely located (less than 10 m apart) sometimes they did not easily share their resources with other unknown people. In our opinion they identified typical problems of *Mobile Ad hoc NETworks* (*MANET*) that still are not efficiently solved in our days. They also have to treat the problem of service disruptions due to *Bluetooth* channel issues.

A cloud can be used to balance opportunistic sensing by observing the proximity of participants and their trajectory [104]. The authors simulated fair scheduling algorithms of sensing operations among the participants. Their objective was to eliminate redundant sensed data and improve energy savings. More work must be done to take into account more complex phone user movements and energy wasted in the communications with the cloud and close phones.

Machine learning algorithms can be used to dynamically predict device energy-efficient consumption [105]. In this case, authors showed that the best results were obtained using neural networks and k-nearest neighbor algorithms.

## 4. Design Aspects of Mobile Sensing Systems

Fusion, learning, security, privacy and energy saving are normally implemented in the phone and they are sometimes coordinated by a sensing server. In this section we will present a holistic view of the design of a MSS that can include a WSN, a cloud and a social network. There are theoretical research works that consider the optimization of the above issues when a WSN is considered for extending the sensing capacity of phones. Other works consider cloud and social network technology. We review these works that have not been included in other surveys and present some new ideas of the design of practical MSS.

**Figure 1.**
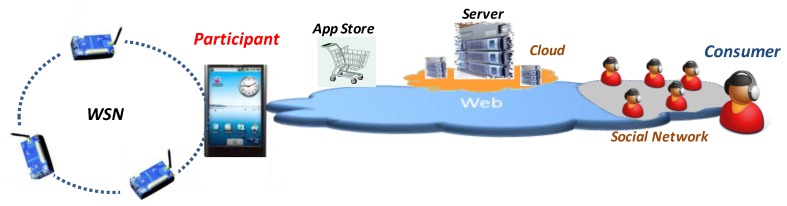
System architecture of a generic MSS.

In Figure 1 we show our MSS schema. The sensor-rich phone executes a sensing mobile *App*. This *App* is downloaded from an *App* store, uses the phone's sensors and can access a WSN using a middleware. Moreover, several geographically near phones can communicate locally their sensing tasks to improve sensing. The WSN is not part of the MSS. The phone can receive data from the coordinator of the WSN, extending the sensing capability. Moreover, the phone can upload sensing instructions to the WSN coordinator. The phone reports data to the server application using Web services. The server always makes the presentation of the sensing results to the consumer. In case the sensing process is organized by an agency, this server will be in charge of *distributing sensing tasks* among the participants (the same applies for crowd MSS). This server can be executed in a machine or it can be executed in several geographically distributed machines in order to distribute the computing load (configured as a *cloud* service). In case the consumers were part of a social network they can use it to share sensed data, processing and visualization.

We propose the following ideas for efficiently implementing sensing, fusion, learning, security, privacy and energy saving:

- *Sensing, fusion and learning*. They can be implemented in the WSN, the phone *App* or the sensing server. In the first case, the manager node of the WSN is in charge of sending the fused data processed by a simple fusion algorithm to the phone *App*. The *App* could implement low computation fusion and learning algorithms in order to save energy. The server can implement complex fusion algorithms. Fusion and learning processes can be distributed efficiently among the WSN, the phone App and the server in order to balance communications and computations. Web services can be used to report data to an Internet sensing server because it is the most used standard in Internet communications in our days.
- *Security and privacy*. Current WSNs allow encrypting sensed data. This mitigates the adverse effects of the security attacks to the WSN. We have reviewed several methods to implement security and privacy in the mobile phone, but some privacy methods are implemented in the server. We propose that the WSN implements encryption, and security and privacy processes be distributed among the *App* and the server. This distribution can be done depending on the power of the phone and the complexity of security and privacy algorithms.
- *Energy saving*. The WSN can reduce the energy consumption of the phone. The server can efficiently distribute the sensing tasks among phones to reduce the work to be done by the phone.
- *Local communication among phones*. Normally, in opportunistic and participatory sensing the phones do not communicate locally. We argue that local communications among phones improves the energy saving and privacy by allowing the efficient distribution of sensing tasks. A mobile cloud technology can be used where relevant.

Next we present some research works that justify our proposals and our identified research challenges.

### 4.1. Web Services, Cloud and Social Network

A server can implement fusion and learning based on the sensed data coming from a phone. For example, Cui *et al.* show in [106] how a server can fuse inputs from different phones with the aim of guiding the design of energy consumption awareness *Apps* to preserve the battery of the phone. The energy consumption awareness *App* makes corrective actions to control the rate, and the sampling duration of phones'sensors. The design of accurate activity and context detection algorithms can be achieved with several Android phones [107] collecting sensing data sets, in several parts of the World, tagged with appropriate ground truth information about the user activity. Context-aware applications can be built using cloud services for visualization and reasoning [108]. They provide a set of tips for optimizing the communication among the application and the Internet cloud. A context oriented programming model proposed that each component, referred to as a Widget, maintain updated information about a specific context [109]. Widgets were allocated in a cloud server or in a mobile device and communicate with each other using standardized ontology for filtering, fusing and/or aggregation of context information. A directory-based service to update information about the overall collection of Widgets was used and applications could read the last updated context accessing that directory. The main barrier to implementation of that proposal is users' mistrust, since the privacy is not guaranteed if the mobile devices would not be under the owner's control.

Web services must coordinate the data gathering (reporting) process from sensors (integrated by a middleware) and communication among consumers using social network software. Normally a MSS uses particular mechanisms for data gathering.

Next we present some recent initiatives to guide the implementation of middleware and Web services appropriate for efficiently reporting sensed data to a server.

A Web service named Orienteer which provides client *Apps* with the orientation of proximate Android devices (∼1.5 m) was presented in [110]. A client *App* has to register the mobile device with the Web service (a Java application) using the Client's Google account (cloud service). That cloud service must determine which client devices were close to the client that requested the Orienteer service.

*SensOrchestra* [111] relied on a server allocated in Internet to infer location by fusing built in sensor data such as audio recordings and images, and a list of nearby phones, coming from mobile phones forming a *Bluetooth ad hoc* network. The server combined the correlated sensor data using a classifier fusion model and sent the recognition results back to the phones.

Web services are often used to report and share sensed data using very simple mechanisms. Some ideas about the implementation of MSS using Web services were presented in [112]. They reviewed requisites, challenges and applications of Web services. We state that a MSS must adapt its communication to the new service architecture of the future Internet [113]. In particular the reporting service (*description*, *discovery*, *access* and *composition*) is the key aspect when designing a MSS. The sensed data must be well described using an ontology or simply an *eXtensible Markup Language* (*XML*) description. Several Web services have been designed with this purpose in mind. For example *REpresentational State Transfer* (*REST*) Web service addresses only basic distributed interaction/coordination by using the standard Web mechanism: any entity on the Web is a resource that has a *Uniform Resource Identification* (*URI*) and can be accessed using the standard *HiperText Transfer Protocol* (*HTTP*) operations. Because *REST*' interface is universal, it can be used by any Web application. It is very simple to program and it could be used by MSS.

Heterogeneity is a challenging topic in sensed data discovery and access. The coordination of the transparent access to heterogeneous data from different sensors using a middleware is presented in [114]. That middleware runs on the WSN and the phone (*Android 2.3*) implementing answers to application requests and monitoring complex data requests of different applications. The sensor manager finds the minimal set of sensors to provide the answer to application requests. The resource coordinator mediates among sensors when concurrent requests are issued to them. The application broker receives the requests from the applications.

Web services must also facilitate the composition of the sensed data. This composition ranges from the simple data integration to the complex computation of the semantic associated to users and sensors [115].

When the Web service allows complex consumer's queries to receive sensed data, the best access service to sensed data will be provided. These Web services must facilitate an orchestration of different sensing applications. The best form to do this is using ontology to decouple the complex queries from the low level details of the underlying sensors [116]. A step beyond this research direction for a people-centric participatory MSS (healthcare) is [117]. It considered a middleware to manage a *Wireless Body Area sensor Network* (*WBAN*). This MSS reported sensed data using *RESTful* which provided interoperability of WBANs with virtual world environments, social networks and other Web 2.0 applications. That middleware used proxies supporting mobility and localization of *Bluetooth* devices in the WBAN.

Web services must allow interoperation and sensed data reuse among different applications in crowd MSS when a lot of heterogeneous and independent sensing *Apps* that re-use sensed data are considered [6]. An additional objective is to implement an intelligent composition system to allow energy saving avoiding rapid phone battery drain. Interoperation, data reuse and energy saving can be implemented in a middleware [118]. Those authors do not specify the technology to be used to build that middleware. We think that *REST* is an appropriate Web service to program this middleware. Moreover, *mashups* can also be used. In a crowd MSS a requester can recruit phone users (or participants) to provide sensor data to be used towards a specific goal or as part of a social or technical experiment [7]. Any non expert requester can start (from his/her phone) a sensing campaign recruiting participants using a simple *App* in which they only must fill an XML file. The authors focus their work on the design of a platform for managing this kind of *Crowd MSS*. Once some participants have been registered (using for example *www.mturk.com/mturk/welcome*), this XML file is sent to a cloud server which processes it. The cloud server executes batch processes for sensing campaign instructions to the participants. Finally, the cloud server communicates the results to the requester.

*Misco* [119] was a mobile cloud composed by peer mobile devices that run mobile sensing and context aware applications and used REST. A mobile cloud computing architecture, like *mCloud* [120], can run resource-intensive applications. The sensing fusion computations were done in a single mobile device or distributed among collections of cooperating phones, tablets and other high computing mobile devices. Some reasons to do the computations locally were communications energy saving using WiFi instead of 3G, and the reduction of the complexity of Internet clouds.

Privacy can be improved if participants will upload sensed data sending them to their friends in a social network instead of sending sensed data directly to the sensing server [88]. These friends could randomly choose another friend. Hopping goes on until some threshold defined by the participant is reached, and then the last user uploads the data to the server.

### 4.2. Local Phones'Communications and Outer WSN

As shown in Section 2, mobile operating systems or other frameworks allow accessing the phone sensors. However, managing an external WSN is difficult, but this allows energy savings if an efficient distribution of sensing tasks among phones and WSN is done [121]. An introduction to the design of software platforms to access external WSNs is provided in [122]. Next, we present some research issues classified by sensing domains.

In the healthcare domain, a *J2ME* platform to manage a service that was aware of users' conditions such as heartbeat, posture, and movement through monitors of physiological signals (for example, electrocardiograph, thermometer, and 3-axis AS) and environmental conditions (for example room temperatures), by communicating with environmental sensors was presented in [122]. Its authors also presented several research issues: (a) communicating to WSN via a mobile sensor router attached to a user's phone; (b) analyzing the sensed data derived from networks by cooperating with sensor middleware on a remote server to capture someone's contexts and (c) providing context-aware services for mobile users. *Amulet* [123] collected users' health information and forwarded it to a health record system in a secure way. It enabled continuous sensing and actuation, requiring a wireless gateway (phone or AP) only for occasional connectivity from the WSN to back-end servers and other off-body network resources.

Another research issue is how to obtain the best partitioning of the sensing geographical field to allocate the WSN nodes and let MSS participants use a *Publish*/*Subscribe* architecture to manage sensor events. In [115], whenever a subscribed event is detected by the WSN, notifications with relevant data were disseminated to the corresponding mobile participants. They simulated three kinds of nodes: brokers (that had a wired connection), sensors and phones. Any wireless sensor or phone can join the network if they can communicate with any of the brokers. The role of the brokers was to maintain the information and process the subscribers' requests to route event notifications and handle the subscribers' handover. This is an initial work proposal, but more complex scenarios of coverage loss must be included. Additional research issues of the partitioning problem are access to the WSN of several mobile users simultaneously and publish the events in a social network [124]. The authors considered that each mobile participant accesses a concrete sensor and this sensor must receive the information of its vicinity. The approach presented in this paper relies on a simple simulated algorithm to let a sensor network self-organize a virtual partitioning in correspondence to spatial regions characterized by similar sensing patterns, and to let distributed aggregation of sensed data take place on a per-region basis. This work discusses a set of problems in the connection between phones and the WSN in order to build a large scale geographical field of sensing. Additional complications of the above scenarios were presented in [125]: mobile participants accessed the WSN disseminated in remote areas where only opportunistic communications can be used. For this reason, it is necessary to implement algorithms that maximize parameters like information utility locality, time-to-live, mobility and user interests. These authors presented theoretical (and simulated) results for optimizing those parameters including the novelty that they allowed the phones to upload data into the sensors in order to let other participants download them. The challenge of data dissemination also appears in opportunistic IoT: That is, the interchange of data between things connected to Internet and phones or *vice versa* [126]. Opportunistic MSS and communication appear due to the coordination among phones and things are dynamic and participants and consumers of information are never being connected at the same time to the same part of the network. For this reason, data stored in things should be moved and replicated in the network. This work relies on the consideration of the most realistic scenarios.

Mobile phones can locally communicate information to infer their localization by fusing position information received from the neighbors with their own position in order to obtain new localization estimation without relying on GPS [127]. The overall consumption decreases with this collaborative GPS localization scheme.

Table 3 summarizes the characteristics of the reviewed systems attending to a set of parameters. We name the system, and we write in the corresponding column whether it uses middleware, social network publication, fusion, cloud, security or energy saving techniques or not. We also name the types of web service and middleware in case we name it.

### 4.3. Social Aspects

We consider two basic stakeholders: the participants and the consumer of the sensed data. Participant and consumer can be the same person (*prosumer*). The participant sometimes acts philanthropically (opportunistic MSS) or belongs to an agency that organizes the sensing campaign (a kind of participatory MSS), or is organized by a simple requester (a kind of crowd MSS). In the last two cases there is another kind of stakeholder that is the responsible to organize the sensing campaign. Nowadays, a lot of organizations are dedicated to sense data for scientific projects. The idea of [128] was to allow a lot of people to register data in a sever eliminating the inconveniences of complex mobile user interfaces. This is a case of crowd sourcing applications with the help of the user that manually registers data in a server eliminating the need to install complex mobile sensing *Apps* to read sensed values and publish them in the server. The relation among the above stakeholders is anonymous, but in some cases there can be other kind of relations, for example a social relation among the participants and the responsible of the agency. Whatever the relationship between stakeholders is, the really important issue is that the participatory MSS must achieve a good acceptance among participants and consumers.

A current important social research challenge in participatory MSS is to study the barriers bound by mobile users when using those systems. Several barriers are [129]: trust, privacy, popularity, difficulty, embarrassment, overload, usefulness, personalization and danger. There are reasons for the adoption of participatory MSS, e.g., when participants find value in comparing their behavior with others [130], typically not from within their close social circle. The behavior of the users can be discovered using a quantitative questionnaire [129], analyzing mobile data to extract phone usage categories [131] and letting the participants to compare their behavior with others [130]. In general the above works state that privacy is the most important social barrier to MSS, and [47] stated that it therefore remains perhaps the most critical obstacle.

### 4.4. Research Challenges

We have presented ideas for designing a MSS. But some of the reviewed works are theoretical or are in their first stage. For that reason, we now present some research challenges:

- More works in the line of [129–131] are needed to better understand other barriers to mobile sensing aside from privacy.
- Simulation of different scenarios was used in [115,124,125]. Experimenting with real scenarios will reveal new unexpected problems with the wireless channel.
- The theoretical work in [115] was a work that was applied to a concrete scenario. Works like this could be put into practice to show real solutions.
- According to [116]: (a) the vast majority of MSS deals with non audio/visual data and do not provide capabilities for searching and processing multimedia data. More effort must be done in order to support audio/visual information; (b) search engines should support ambient intelligent synthesis in real time for environment-centric MSS; (c) these engines could support the dynamism of sensed data and must consider the sensed data stored in social networks.
- A recently proposed protocol for device to device communication is the *Constrained Application Protocol* (*CoAP*) [132,133]. The evolution of this draft should be taken into account for the implementation of future MSS when communicating with a WSN.
- Security and privacy are challenging research issues in MSS. A practical implementation of [88] should consider the problems of hopping communications among the phones due to the interruption of wireless channels in multi-hopping networks. Moreover, in our days hopping direct communications among commercial phones are only allowed in a few phone brands (and models).

## 5. Conclusions

The aim of this paper is to present a holistic vision of mobile sensing systems. We have reviewed a large amount of research works and sensing applications and analyzed design issues and key aspects to design mobile sensing systems. Moreover, we have summarized the observed trends in those research works.

Two initial conclusions are: the majority of sensing applications are designed for *Android* and very few research works used social networks to exploit the relationship of friendship among the participants of a sensing campaign.

We have reviewed the variety of sensors and phone software for managing sensors. External sensors usually accessed by phones are even more varied. We showed that one inconvenient for MSS is that the sensors and software are specific to each phone, but the software to manage sensors must be interoperable and allow multitasking to produce efficient interoperable mobile sensing systems. Despite several initiatives such as *JSR*, *Khronos* and *PhoneGap*, this is not currently a reality.

Perhaps privacy is the most important social barrier that limits the acceptation of mobile sensing. Mathematical fusion models have been formulated to consider sensing data that are not correlated, but these methods must be implemented in practical mobile sensing systems. For example, currently some phones include a trusted computing platform that must be taken into account for security implementation.

Energy saving is a critical technical barrier. Theoretical works must be implemented in practical mobile sensing systems. Middleware also must be developed to show its power in practical mobile sensing systems. Utilization of multiple radios switching could help phones to save energy. For participatory and opportunistic mobile sensing systems an economic incentive must be considered to stimulate the participants.

The efficient implementation of mobile sensing systems is hard. We finally present some learnings:

- Sensor fusion (producing software sensors) is still in its infancy. Learning must be used to empower mobile sensing systems.
- Healthcare external sensors may be included in the long term in phones.
- Communications between phones and wireless sensor networks (or single external sensors or other phones) must overcome the present limitations of *Bluetooth*, *WiFi Direct* or *WiFi Tethering* technologies to form efficient multihop WiFi (or other technology) communication networks. Research trends on machine to machine communications could improve the current *ad hoc* communication among phones.
- The middleware to access sensors in the wireless sensor network may fuse different physical sensors values in order to provide high level semantic information for context awareness applications. It must be also be cross-platform and manage multitasking applications in the phone.
- Consumers should be enabled to subscribe to sensing event campaigns in order to receive alarms when appropriate.

## Figures and Tables

**Table 1.** Examples of APIs for managing phones' sensors.

***BlackBerry***	***AccelerometerData*** and ***AccelerometerSensor***	AS readings. AS changes can be detected using the interface *AccelerometerListener*.

	***GPSSettings*** and ***GPSInfo***	They provide access to the GPS.

	***TouchEvent***, ***TouchGesture*** and ***Touchscreen***	*TouchEvent* contains touch input events originating from the user. *TouchGesture* detects gestures. Touchscreen provides low-level access to the touch screen.

***Windows***™***Mobile***®	***Microsoft.Devices.Sensors***	AS, CS, and GS readings. *Motion* class handles the low level sensor calculation and allows applications to obtain the device' sattitude, rotational and linear acceleration. Other classes are: *Accelerometer, Compass and Gyroscope*.

	***System.Device.Location***	It exports the Windows Phone Location Service API enabling the development of location-aware applications.

***Symbian***	***Sensors***	API for receiving events from AS, ALS, CS, GS, PS, light, magnetometer, orientation, rotation, and tap sensors.

	***Location***	API that gives users the capability to develop location-aware applications.

	***Multimedia***	API that gives developers a simplified way to use audio and video playback, and access IS functionality.

***Android***	***SensorManager***	Class to access and list sensors (*getSensorList()* method), register sensor event listeners (*registerListener()*), and acquire orientation information.

	***Sensor***	Class representing a specific sensor (some methods: *getResolution()*, *getPower()*, *getMinDelay()* and *getMaximumRange()*).

	***SensorEvent***	Class to create a sensor event object to know the type of sensor that generated the event, the accuracy of the data, and the time at which the event happened.

	***SensorEventListener***	Interface to create two callback methods that receives notifications (sensor events) when sensor values change or when sensor accuracy changes.

***iOS***	***CMMotionManager***, ***CMAccelerometerData***, ***CMAttitude***, ***CMDeviceMotion***, ***CMGyroData*** and ***CMMagnetometerData***	*CMMotionManager* for AS, magnetometer and GS readings. Processing data of attitude, rotation rate, calibrated magnetic fields, the direction of gravity, and the acceleration. *CMAccelerometerData* represents an AS event. *CMAttitude* offers three different representations of attitude. *CMDeviceMotion* to know values of the attitude, rotation rate, and acceleration of a device. *CMGyroData* contains a single measurement of the device's rotation rate. *CMMagnetometerData* which encapsulates measurements of the magnetic field made by the device's magnetometer.

**Table 2.** Brief summary of the analyzed works that use fusion.

**Reference**	**Application** (a)**/App Developers** (d)	**Mobile OS**	**Sensors**	**Detailed Fusion Algorithm**	**Application Domain**
[35]	a	Android	WiFi	No	Place-centric sensing
[10]	a	Android	Microphone	Yes (1)	Crowd sensing
[36]	d	Android		Yes	Place-centric sensing
[26]	a	Android	IS and AS	Yes	Urban sensing
[74]	a	Maemo 5	Motion sensors	Yes (2)	Indoor Navigation System
[78]	a	Symbian	Microphone and AS	Yes (3)	Activities of daily living
[75]	Work in progress	Maemo 5	At least one position sensor (GPS, AS)	Yes	Indoor Navigation System
[24]	a/d	Windows Mobile 6.5 (emulator)	Microphone	Yes	Healthy sleep
[31]	d	-	GPS, IS and AS	Yes (4)	Classification (for example user location and activity)
[76]	a	Android and iOS	Digital CS and IS	Yes	Orientation in indoor environments
[28]	a/d	Windows Mobile	Speaker, microphone and communication sensor	Yes	High-accuracy ranging and localization
[29]	a	Android	GPS	Yes (5)	Localization
[25]	a/d	iOS	Microphone	Yes	Sound classification
[30]	a	Symbian	AS	Yes	Mobile data segmentation
[79]	General Framework	Not specified	AS and WiFi	Yes (6)	Human behavior pattern analysis
[39]	a	iOS	Outer CO sensor	Uses commercial services	Urban sensing

(1) Machine learning algorithms;

(2) Dead reckoning technique;

(3) Hidden Markov model;

(4) Commonsense contextual reasoning;

(5) Semantic location;

(6) Classifier fusion model.

**Table 3.** Brief summary of the reviewed MSS characteristics.

**System**	**Middleware**	**Web Service**	**Social Network**	**Fusion**	**Cloud**	**Security**	**Energy Saving**
[42]	Yes	No	No	Yes	No	No	No
[123](4)	Yes	Yes	No	Yes	No	Yes	No
[32]	No	Yes (1)	No	Yes	No	Yes	Yes
[41]	Yes (2)	Yes	No	Yes	No	Yes	No
[26]	No	No	No	Yes	No	No	No (4)
[40]	Yes (3)	No	No	Yes	No	No	No
[110]		Yes (5)	No	Yes	Yes	No (4)	No (4)
[34]	No	No	No	Yes	Yes (6)	No	Yes
[121]	Yes	No	No	Yes	No	No	Yes
[38]	No	No	No	Yes	No	No	No

(1) The services of the proxy layer are required to provide a REST interface and assume XML creation and parsing responsibilities;

(2) Object oriented middleware for distributed systems;

(3) Web server between the client and two databases: 3D Map and Information Database;

(4) In progress;

(5) *Orienteer* web-service;

(6) The bus arrival time prediction service can be implemented in a computing cloud.
